# Editorial: Cognition in Mood Disorders

**DOI:** 10.3389/fpsyt.2019.01013

**Published:** 2020-01-31

**Authors:** Mayowa Oyesanya, Catherine J. Harmer, Allan H. Young

**Affiliations:** ^1^ Department of Psychopharmacology and Emotional Research, Department of Psychiatry, Warneford Hospital, University of Oxford, Oxford, United Kingdom; ^2^ Oxford Health NHS Foundation Trust, Warneford Hospital, Oxford, United Kingdom; ^3^ Department of Psychological Medicine, Institute of Psychiatry, Psychology and Neuroscience, King's College London & South London and Maudsley NHS Foundation Trust, Bethlem Royal Hospital, Beckenham, United Kingdom

**Keywords:** cognition, depression, mood disorders, cognitive biomarker, anxiety

## Introduction

Mood disorders are common, complex, and one of the main causes of morbidity worldwide (1). There has been an increasing recognition that cognitive dysfunction is a central aspect of most mood disorders, as well as being closely related to the functional impairment that these disorders commonly cause (2, 3). Therefore, appropriate assessment and management of cognitive impairment(s) in mood disorders is important for the optimal treatment of these disorders more broadly. Research in these areas is ongoing and has the potential to improve our understanding of the neurobiological and neuropsychological mechanisms underpinning cognitive dysfunction in affective illness. In addition, developing tools to measure cognitive deficits more objectively, may augment the diagnosis of affective disorder and support current, and future efforts, to improve the classification of psychological symptoms and processes in psychiatry (4). This could allow for the identification of patterns of cognitive deficits which may be more amenable to certain treatments or may be of prognostic utility.

In this editorial, we seek to summarize and organize the research literature published in this special Research Topic — Cognition in Mood Disorders. In this special edition, research papers published within this topic will be discussed within the following headings: the neurobiology of cognition, experimental models for understanding cognition, potential predictive cognitive markers, and the assessment and management of cognitive dysfunction, in mood disorders.

## Neurobiology of Cognitive Dysfunction in Mood Disorder

This special issue includes a focus on the neurobiological underpinnings of cognitive impairment in different mood disorders. King et al. explored the relationship between neuroinflammatory processes, dysfunction in glutamate neurotransmission, and subsequent cognitive deficits in depression (with a focus on learning and memory). Magnetic resonance spectroscopy of the anterior cingulate cortices of a group of patients with bipolar II disorder and healthy controls, found no difference in anterior cingulate glutamate or inflammatory markers; although poor performance on one of the cognitive tasks, was predictive of a poorer response to psychological therapy. The study was a pilot and generated key hypotheses for future higher-powered studies.

Gao et al. investigated differences in the functioning of the default mode and executive control networks in depressed patients versus a group of controls, using resting state functional magnetic resonance imaging (rs-MRI). A treatment naïve group was chosen to eliminate any potential confounding effect of antidepressant use on neural response. These depressed participants were found to have lower and higher network homogeneity (NH) in different parts of the default mode network. They were also found to have reduced executive function compared to controls. These finding suggest that changes in executive function and the default mode network occur independently of treatment effects in depression.

Wang et al. used transcranial doppler and 320 slice- computed tomography (CT) scanning to measure regional cerebral blood flow velocity and regional cerebral blood flow respectively, in manic patients compared to a group of patients with major depression and healthy volunteers. The authors explain that although such whole brain perfusion scanning using CT has seen use in cerebrovascular disease, its use in psychiatry is relatively novel. Regional cerebral blood flow and velocity was increased in the left medial temporal lobe and the right hippocampus in manic patients compared to the other groups. As the authors state, it would have been of additional interest to have a bipolar depression group as a comparison group in their study. Advances in neuroimaging, as shown in Wang et al.'s study and the others in this issue, have allowed for more rigorous and quantitative assessment of previously uncharted areas of cognition in psychiatry.

### Neuropsychological Experimental Models for Understanding Cognition

The studies summarised in this section have used neuropsychological experiments involving healthy participants and/or participants with affective illnesses to analyse specific aspects of cognition of relevance to affective disorders.

Walsh et al. and Chase et al. studied reward processing in healthy volunteers and in bipolar disorder respectively. In Walsh et al.'s study, the administration of a single dose of bupropion (a noradrenaline and dopamine reuptake inhibitor) led to statistically significant differences in emotional processing, but not reward processing, compared to placebo. Using a cued reinforcement reaction time task, Chase et al. found all groups (depressed bipolar, euthymic bipolar, unipolar depression and healthy controls), showed similar reaction time performance in the task, although the euthymic bipolar group showed an increase in commission error rate in high reward conditions. The results of the study provided some evidence for response-calibration deficits that were specific to patients with bipolar disorder.

The other three studies in this section sought to explore the relationship between depressive symptom burden and metacognition (Payne et al.), cognitive control of emotional conflict in clinical versus varying degrees of trait anxiety (Yu et al.) and the association between social cognition and paranoia (Savulich et al.). Payne et al. provide evidence of an association between increasing depressive symptom burden, a greater tendency to adjust levels of confidence in response to new evidence, and lower overall confidence levels. They point to the need in their study to replicate their findings with a sample of patients with clinical depression. Yu et al. demonstrated that deficits in cognitive control of emotional conflict were present in individuals with high trait anxiety, with more severe deficits occurring in generalised anxiety disorder. They hypothesized that trait anxiety may produce these impairments, potentially leading to clinically significant levels of anxiety. Savulich et al. showed that paranoid thinking may reduce cooperation in the pursuit of mutual reward and that delusional ideations can predict maladaptive feelings of shame when experiencing interpersonal harm in a moral emotional processing task.

As these studies have shown, experimental neuropsychological experimental models are important in forming and testing hypotheses about relationships between cognition and other symptom domains that may mediate the relationship between mood disorder and subsequent cognitive impairment.

### Potential Predictive and Diagnostic Cognitive Markers in Mood Disorders

Two further articles review the literature to assess the potential of cognitive deficits as predictors of treatment response in depression (Groves et al.) or as intermediate diagnostic phenotypes in bipolar disorder (Kessing and Miskowiak). Evidence from the review by Groves et al. is mixed. Some studies in the review demonstrated an association between poorer baseline cognitive functioning (particularly in executive function and attention) and poorer treatment response, although other studies failed to find this, and this association was also affected by the treatment used (different antidepressants, antidepressant and psychotherapy etc). Due to the methodological heterogeneity of studies included in the Groves et al. review, a qualitative synthesis of these studies was performed. Kessing and Miskowiak concluded there was not enough evidence that cognitive deficits in bipolar disorder were sufficiently specific to the disorder, to serve as useful intermediate diagnostic phenotypes. Therefore, more research is needed to see if cognitive impairments specific to bipolar disorder can be identified.

Smirnova et al. presented interesting data which suggests that linguistic analysis of information produced by healthy participants or participants with mild depression and “normal sadness”, can distinguish between these three groups. Participants with mild depression tended to produce longer responses, with their written responses presented more in a narrative than analytical manner compared to healthy controls. Participants with mild depression also tended to use more colloquialisms, tautologies and single clause sentences compared to healthy controls.

Using a prospective study design over a period of 2.5 years, Ruhe et al. investigated whether specific biases in emotional processing, remained in patients with remitted recurrent depression, and whether these deficits could predict illness recurrence. They found that compared to the study control group, such patients had persisting negative attentional biases toward faces and self-relevant characteristics with a negative valence, and tended to misclassify neutral faces as expressing anger or disgust. These differences were not predictive of future depressive recurrence.

### Assessment and Treatment of Cognitive Dysfunction in Mood Disorders

The final section of this article outlines the articles which may be the closest to direct clinical translation, as they concern the evidence for the assessment and management of cognitive dysfunction in depression (Zuckerman et al.; Fiorillo et al.), schizoaffective disorder (Lopez-Fernandez et al.) and a trial protocol comparing personalised vs standard therapy for the treatment of cognitive dysfunction in depression (Knight et al.)

Zuckerman et al. emphasize the lack of any gold standard test of cognitive impairment in major depression and the unsuitability of current assessment measures to measure it (e.g., Hamilton-Depression Rating Scale). The use of cognitive behavioural therapy, cognitive remediation therapy, pharmacotherapy and neurostimulation techniques (e.g., transcranial magnetic stimulation), were also outlined in this review. On the other hand, Fiorillo et al. focus on the available validated tools for assessing objective and subjective cognitive dysfunction major depression, such as the Screen for Cognitive Impairment in Psychiatry-Depression (SCIP-D – a measures objective cognitive dysfunction) and the Cognitive complaints in Bipolar Disorder Rating Assessment (“COBRA” – a measure of subjective aspects of cognitive dysfunction) (5).They also outline the need to move towards achieving full recovery of function as the main therapeutic target in major depression, rather than just clinical remission.

Lopez-Fernandez et al. performed a systematic review on the effectiveness of cognitive remediation therapies in schizoaffective disorder, finding some evidence that they can be effective in improving social cognition and neurocognition. These conclusions were however limited by the relatively small of studies available for review and their design limitations.

Knight et al’s. proposed clinical trial will finish recruiting patients in 2019 and aims to compare a personalised therapeutic regime targeting cognitive impairment, social cognition, and emotional processing problems, to standard treatment for each of these domains. The personalized therapy will be more tailored and intensive; i.e., a greater number of sessions will be assigned to areas of cognitive dysfunction which were more marked at baseline assessment.

## Conclusion

The research presented in this issue reflects the diversity and depth of the research into cognition in mood disorders. The exciting articles in this Research Topic give a real insight into the various methodological approaches being used in this research area to illuminate mechanisms, treatment targets, and clinical translation of work in the area of cognition in mood disorders. For example, one promising area of translation is the use of cognitive test batteries to predict treatment in identifying earlier treatment response to depression, and work on this is ongoing (6). Other future areas could include the integration of cognitive markers into systems of diagnostic classification (such as future revisions of the DSM-V and ICD-11), or to develop personalised treatment regimes, developed to effectively address specific cognitive deficits. The current articles provide a fantastic primer in an area whose significance is increasingly evident, and which is developing in new and exciting directions.

## Author Contributions

MO wrote the first draft of the manuscript. MO, CH, and AY contributed to manuscript revision, read and approved the submitted version.

## Conflict of Interest

The authors declare that the research was conducted in the absence of any commercial or financial relationships that could be construed as a potential conflict of interest.

The handling editor declared a past collaboration with one of the authors MO.
